# Total Anomalous Pulmonary Venous Connection with Lethal Pulmonary Venous Obstruction Managed by Multidisciplinary Cooperation

**DOI:** 10.1155/2021/6619458

**Published:** 2021-01-15

**Authors:** Kana Ito, Ayako Chida-Nagai, Osamu Sasaki, Nobuyasu Kato, Takeshi Umazume, Satoshi Kawaguchi, Kazutoshi Cho, Gaku Izumi, Hirokuni Yamazawa, Atsuhito Takeda

**Affiliations:** ^1^Department of Pediatrics, Hokkaido University Hospital, Sapporo, Hokkaido 060-8648, Japan; ^2^Department of Pediatrics, National Defense Medical College, Tokorozawa 359-8513, Japan; ^3^Department of Cardiovascular and Thoracic Surgery, Faculty of Medicine and Graduate School of Medicine, Hokkaido University, Sapporo, Hokkaido 060-8648, Japan; ^4^Department of Obstetrics and Gynecology, Hokkaido University Hospital, Sapporo, Hokkaido 060-8648, Japan; ^5^Maternity and Perinatal Care Center, Hokkaido University Hospital, Sapporo 060-8648, Japan

## Abstract

**Background:**

Total anomalous pulmonary venous connection (TAPVC) is a critical congenital heart disease for which emergency surgery is required after birth. In cases of no intervention, TAPVC is associated with a high mortality rate in the first year of life. Although foetal echocardiographic techniques for diagnosing TAPVC have improved, TAPVC remains one of the most difficult congenital heart diseases to diagnose via foetal echocardiography. Here, we report a case of TAPVC with pulmonary venous obstruction (PVO), which was diagnosed via foetal echocardiography. *Case Presentation*. On foetal echocardiography at 32 weeks' gestation, a large atrial septal defect, enlarged superior vena cava, and continuous flow pattern in the vertical vein from the common chamber were observed in the foetus. Paediatric cardiologists and cardiac surgeons, neonatologists, and obstetricians planned to perform a caesarean section and emergency heart surgery. The male infant was born at 37 weeks' gestation via caesarean section, and postnatal echocardiography revealed PVO at the confluence of the superior vena cava and common chamber. Similarly, chest computed tomography confirmed the foetal diagnosis. The postnatal diagnoses were TAPVC type Ib, PVO, atrial septal defect, and patent ductus arteriosus. Surgical repair of the TAPVC was initiated within the first 3 hours of life. Screening brain echocardiography and head computed tomography revealed intracranial haemorrhage and hydrocephalus. Therefore, the patient underwent emergency bilateral external drainage on day 13. On day 48, a ventriculoperitoneal shunt was inserted owing to progressive brain ventricular dilatation. The patient was discharged home on postoperative day 68.

**Conclusions:**

Although the prognosis of TAPVC with PVO remains poor, continuous observation through foetal echocardiography and early interdepartmental collaboration can result in good outcomes.

## 1. Introduction

Total anomalous pulmonary venous connection (TAPVC) is a critical congenital heart disease for which emergency surgery is required after birth. The detection and management of prenatal-isolated TAPVC remain challenging [1]. Here, we report a case of TAPVC with pulmonary venous obstruction (PVO), which was diagnosed via foetal echocardiography and managed with emergency surgery after birth.

## 2. Case Presentation

A 33-year-old primigravid woman underwent foetal echocardiography at 32 weeks' gestation at a local hospital. The obstetrician suspected a combination of TAPVC and PVO and referred her to our hospital.

**Figure 1:**
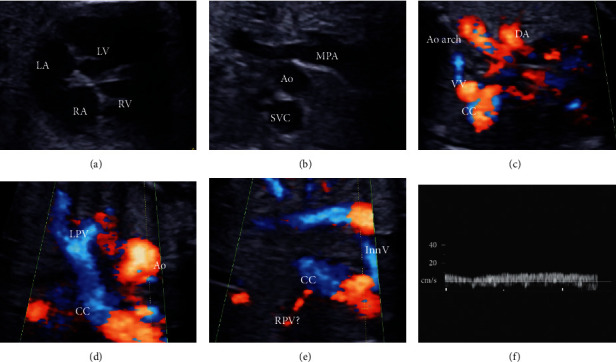
Foetal echocardiography. (a) An atrial septal defect is detected. (b) The superior vena cava is enlarged. (c) Vertical vein (VV) flow from the common chamber (CC) shows a continuous pattern. (d) Left pulmonary vein (LPV) flows into the enlarged CC. (e) The right PV (RPV) seems to flow to the CC. (f) The RPV shows a continuous flow pattern. The A-dip of the flow is seen to disappear. Ao, ascending aorta; Ao arch, aortic arch; DA, ductus arteriosus; InnV, innominate vein; LA, left atrium; LV, left ventricle; MPA, main pulmonary artery; RA, right atrium; RV, right ventricle.

Foetal echocardiography revealed a large atrial septal defect, enlarged superior vena cava (SVC), and continuous flow pattern in the vertical vein from the common chamber (CC) (Figures 1(a)–1(c)). Left pulmonary veins (PVs) drained into the enlarged CC (Figure 1(d)); however, the confluence of the right PV with the CC and SVC was unclear (Figure 1(e)). The provisional diagnosis was a supracardiac-type TAPVC (type Ib according to the Darling classification) with severe PVO from the right PV flow pattern (Figure 1(f)).

Paediatric cardiologists and cardiac surgeons, neonatologists, and obstetricians planned to perform a caesarean section and emergency heart surgery. Prior to the caesarean section, a simulation was performed by each specialist in the operating room.

**Figure 2:**
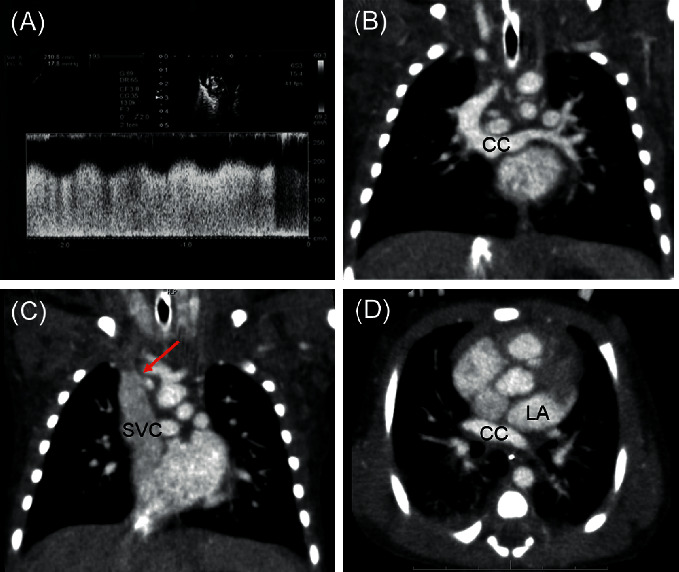
Various tests after birth. (A) Echocardiography displays a continuous flow pattern at the confluence of the pulmonary vein common chamber with the superior vena cava. (B) Contrast-enhanced computed tomography reveals bilateral pulmonary veins flowing into the common chamber. (C) The red arrow indicates severe stenosis of the confluence of the pulmonary vein common chamber with the superior vena cava. (D) The common chamber did not connect with the left atrium.

**Figure 3:**
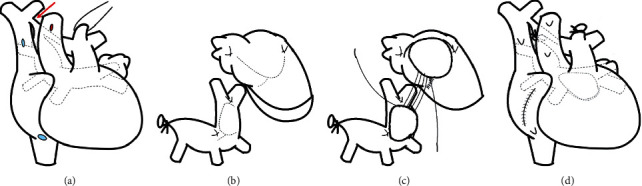
Details of the total anomalous pulmonary venous connection repair. (a) Overall view before surgery. The dotted line shows the pulmonary vein and common chamber. The red arrow shows the stenosis point of the vertical vein. Cardiopulmonary bypass was established with bicaval drainage (blue dots) and ascending aortic cannulation (red dot). (b) Creating the U-shaped flaps at the left atrium and common chamber. (c) Anastomosis of the left atrium and common chamber. (d) Overall view after surgery.

A male infant was born at 37 weeks' gestation via caesarean section. His percutaneous oxygen saturation (SpO_2_) was 60–70%, and venous blood gas analysis revealed a pH of 7.251, pCO_2_ level of 47.5 mmHg, HCO_3_^−^ level of 20.2 mol/L, and base excess of −6.9 mol/L. Bag-mask ventilation improved the SpO_2_ level to 80%, and postnatal echocardiography revealed PVO at the confluence of the SVC and CC (Figure 2(a)). Similarly, chest computed tomography (CT) confirmed the foetal diagnosis (Figures 2(b)–2(d)). The respiratory and circulatory conditions did not worsen with transfer to the CT room, and the postnatal diagnoses were TAPVC type Ib, PVO, atrial septal defect, and patent ductus arteriosus. Severe PVO had been observed at the foetal stage, and postnatal echocardiography and cardiac CT showed findings consistent with those observed on foetal echocardiography. Considering the possibility of respiratory failure due to pulmonary congestion soon after birth, immediate surgical intervention was considered necessary; therefore, tracheal intubation was performed shortly after cardiac CT (synchronised intermittent mandatory ventilation mode: fraction of inspired oxygen, 1.0; positive end-expiratory pressure (PEEP), 3 mmHg; pressure support (over PEEP), 8 mmHg; and respiratory rate, 20 breaths/min). Surgical repair of the TAPVC was initiated within the first three hours of life (Figure 3(a)). To provide a sufficiently wide anastomotic diameter without excessive tension, we made U-shaped incisions on the CC and left atrium (Figure 3(b)) and anastomosed them with the flaps facing each other (Figures 3(c) and 3(d)). Atrial septal defect closure and patent ductus arteriosus ligation were similarly performed. The patient's postoperative haemodynamics were stable, and he was extubated on day six and left the intensive care unit on day seven.

**Figure 4:**
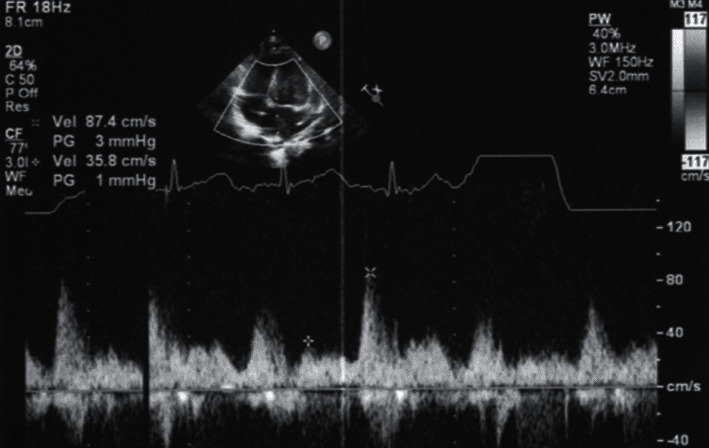
Echocardiography at the age of 4 months in the outpatient clinic. Inflow of blood from the common chamber to the left atrium can be observed. The flow pattern is pulsatile, and no anastomotic stenosis is observed. The cardiac contractility is good, and no other abnormal findings, including pulmonary vein stenosis, are observed.

However, screening brain echocardiography and head CT revealed intracranial haemorrhage and hydrocephalus. Therefore, the patient underwent emergency bilateral external drainage on day 13. On day 48, a ventriculoperitoneal shunt was inserted due to progressive brain ventricular dilatation, and he was discharged home on postoperative day 68. At the time of writing, he was 10 months old. There have been no abnormalities in his growth, development, or haemodynamics (Figure 4).

## 3. Discussion

We reported a case of a prenatally diagnosed TAPVC with a critical PVO that was managed through multidisciplinary collaboration. The patient showed medium-term survival without postoperative haemodynamic problems.

TAPVC without intervention is associated with a high mortality rate (about 80%) in the first year of life [2]. In addition, obstructions of pulmonary venous drainage can be merged into TAPVC and lead to profound cyanosis and respiratory failure presenting with critical illness in the first several hours of life. Although foetal echocardiographic techniques for diagnosing TAPVC have improved [3], TAPVC remains one of the most difficult congenital heart diseases to diagnose via foetal echocardiography. Laux et al. reported that an expert cardiac sonographer detected only 11% of isolated TAPVC cases [4].

To diagnose TAPVC in the foetal period, a single perfect indicator for the detection of isolated TAPVC seems infeasible, as it is important to combine several indicators, such as the presence of a left atrial septum, SVC, innominate vein, or coronary sinus enlargement and abnormal abdominal vessels.

Diagnosing PVO at the foetal stage is challenging because there is lower pulmonary blood flow in the foetal circulation [1]. It is important to check the peripheral PV flow pattern to detect PVO. Normally, the A-dip (cessation of flow or small A-wave reversal during atrial systole) is present in the peripheral PV flow [5] but disappears during PVO, resulting in a continuous flow pattern. As PVs with downstream obstruction demonstrate a continuous low-velocity pattern [6], the PV flow pattern can be easily identified when the colour range of echoes is reduced.

In this case, a U-shaped flap was created for the left atrium and CC to provide a sufficient diameter at the anastomotic site without excessive tension. We expected that this method would prevent PVO recurrence at the anastomotic site.

In this case, a prenatal diagnosis of TAPVC with PVO contributed to the rapid surgical repair and good postoperative course. The experts played their roles from the foetal stage to birth. We opted for a scheduled caesarean section to save the infant. Xiang et al. suggested that a caesarean section was an important predictor of mortality due to TAPVC [7]. However, it is difficult to keep all professionals involved and prepared at all times for an unpredictable normal vaginal delivery. Thus, our choice was reasonable from this perspective.

Our patient developed serious complications, including intracerebral haemorrhage and hydrocephalus. In addition to the vulnerable cerebral vasculature and inadequate autoregulation, the immature haemostatic coagulation system in newborns and the use of heparin in cardiopulmonary bypass increase the risk of intracranial haemorrhage [8]. In addition, we cannot deny the possibility that the use of contrast media immediately after birth may have been a contributing factor for intracranial haemorrhage. However, using chest contrast-enhanced CT, we were able to understand the accurate positioning of the PVs, the common pulmonary vein chamber, and the left atrium in three dimensions. This enabled us to devise a method to suture the common pulmonary vein and the left atrium, using U-shaped flaps, and perform a successful radical surgery. It is exceedingly difficult to determine this positional relationship exclusively by echocardiography. Based on the fact that PVO recurrence has not occurred yet, chest contrast-enhanced CT may be a useful method in such cases.

It is extremely challenging to determine the optimal timing of surgical intervention when there are concerns about the exacerbation of severe PVO observed at the foetal stage. To increase survival rates and reduce postoperative complications, advances in prenatal diagnosis and determination of an optimal surgery timing are crucial. We believe that further studies are necessary to validate the study findings.

This report described TAPVC with severe PVO in a male infant. In-depth foetal echocardiography, preliminary consultation by experts, and tailored intracardiac surgery were helpful in resolving such a severe case of congenital heart disease.

## Data Availability

No data were used to support the findings of this study.
